# DNA Methylation Status of Epithelial-Mesenchymal Transition (EMT) - Related Genes Is Associated with Severe Clinical Phenotypes in Ulcerative Colitis (UC)

**DOI:** 10.1371/journal.pone.0107947

**Published:** 2014-10-10

**Authors:** Tomomitsu Tahara, Tomoyuki Shibata, Masaaki Okubo, Takamitsu Ishizuka, Masakatsu Nakamura, Mitsuo Nagasaka, Yoshihito Nakagawa, Naoki Ohmiya, Tomiyasu Arisawa, Ichiro Hirata

**Affiliations:** 1 Department of Gastroenterology, Fujita Health University School of Medicine, Toyoake, Japan; 2 Department of Gastroenterology, Kanazawa Medical University, Ishikawa, Japan; Institute of Pathology, Germany

## Abstract

**Background:**

Epithelial-to-mesenchymal transition (EMT) is a phenomenon that allows the conversion of adherent epithelial cells to a mesenchymal cell phenotype, which enhances migratory capacity and invasiveness. Recent studies have suggested that EMT contributes to the pathogenesis of ulcerative colitis (UC). We investigated the promoter DNA methylation status of EMT-related genes in the colonic mucosa in UC.

**Methods:**

Colonic biopsies were obtained from the rectal inflammatory mucosa of 86 UC patients and the non-inflammatory proximal colonic mucosa of 10 paired patients. Bisulfite pyrosequencing was used to quantify the methylation of 5 candidate CpG island promoters (*NEUROG1*, *CDX1*, *miR-1247*, *CDH1*, and *CDH13*) and *LINE1*.

**Results:**

Using an unsupervised hierarchical clustering analysis, inflamed rectal mucosa was well separated from mucosa that appeared normal. The *CDH1* and *CDH13* promoters were significantly associated with patient age (*p* = 0.04, 0.03, respectively). A similar trend was found between those genes and the duration of disease (*CDH1*: *p* = 0.07, *CDH13*: *p* = 0.0002, mean of both: *p*<0.00001). Several positive associations were found between hypermethylation and severe clinical phenotypes (*CDX1* and *miR-1247* and a refractory phenotype: *p* = 0.04 and 0.006, respectively. *miR-1247* and *CDH1* hyper methylation and a more severe Mayo endoscopic subscore: *miR-1247*: *p* = 0.0008, *CDH1*: *p* = 0.03, mean of both: *p* = 0.003). When the severe clinical phenotype was defined as having any of five phenotypes (hospitalized more than twice, highest Mayo endoscopic subscore, steroid dependence, refractory, or a history of surgery) *miR-1247* hypermethylation was associated with the same phenotype (*p* = 0.008).

**Conclusions:**

Our data suggest that variability in the methylation status of EMT-related genes is associated with more severe clinical phenotypes in UC.

## Introduction

Ulcerative colitis (UC) affects the colon and rectum and involves the innermost lining mucosa, manifesting as continuous areas of inflammation, with no segments of normal mucosa [1]. It is characterized by chronic and relapsing colonic inflammation of unknown etiology. UC is a multifactorial, heterogeneous, polygenic disease and is precipitated by a complex interaction of environmental, genetic, and immunological factors [2]. The factors explaining the heterogeneity of this disease have remained largely unidentified, and accurate biomarkers to predict disease severity are also limited.

DNA methylation, particularly promoter CpG island hypermethylation, contributes to carcinogenesis [3]. Subsequent studies have also revealed that CpG island hypermethylation also occurs in aged or inflamed tissue [3]–[5]. A high degree of CpG island hypermethylation is observed in both inflamed colonic tissues and dysplastic lesions, suggesting the potential usefulness of DNA methylation as a biomarker for predicting disease severity and cancer risk [6]–[8].

Epithelial-mesenchymal transition (EMT) is a biological phenomenon allowing polarized epithelial cells, which are normally bound to each other within an organized tissue, to transition into independent fibroblastic cells possessing migratory properties and the ability to invade the extracellular matrix [9]–[11]. It has been shown that EMT is involved in the initial step, that is, the acquisition of migratory and invasive capability, in carcinogenesis [12]. Recent studies have also suggested that EMT contributes to the pathogenesis of inflammatory bowel disease (IBD), based on a clinical analysis and animal experiments [13], [14]. Indeed, *CDH1* encoding the epithelial cell marker E-cadherin is known to be reduced in areas of active ulcerative colitis [15]. Promoter hyper methylation of *CDH1* has been shown in inflamed colonic mucosa in UC [16]. A recent genome-wide association scan also identified *CDH1* as a susceptibility locus for UC [17]. Moreover, *CDH13*, encoding another cadherin protein H-cadherin has been shown to be methylated in colorectal cancer [18], an established complication of longstanding ulcerative colitis [19].

A recent genome-scale methylation analysis using an EMT model with ectopic expression of the transcription factor Twist1 in immortalized human mammary epithelial cells identified a number of methylated promoter CpG islands [20]. Because of the importance of DNA methylation and EMT in UC, we hypothesized that epigenetic modification of EMT-related genes may be a pathogenic mechanism in UC. To evaluate the importance of DNA methylation in EMT-related genes in UC, we investigated the promoter DNA methylation status of EMT-related genes in non-neoplastic rectal colonic mucosa of UC and assessed its relationship with various clinical phenotypes.

## Methods

### Ethics statement

This study was approved by the Human Research Ethics Committee of the Fujita Health University School of Medicine. Each participant provided written informed consent for the clinical and laboratory data to be used and published for research purposes. The study was conducted according to the principles outlined in the Declaration of Helsinki.

### Study population

Enrolled in the study were 86 patients with ulcerative colitis (men: n = 48, women: n = 38). The median age was 35 years, and the median clinical duration was 5.5 years. Six cases underwent surgery due to a toxic megacolon or cancer occurrence. Rectal inflammatory mucosal specimens were obtained from all the patients during colonoscopic biopsies and preserved at −80°C until use. Paired non-inflammatory proximal colonic mucosae were also collected from 10 cases. All UC cases were clinically in remission at the time of endoscopy. The histopathological examinations showed mild or moderate inflammation but no evidence of dysplasia or neoplasia at all the sites where the biopsies were taken. Based on appearance during endoscopy, 20 patients showed inflammatory mucosa only in the rectum, and 38 patients showed an extension of the inflammatory mucosa into the left side of the colon (sigmoid and descending colons). The remaining 38 patients showed an extension of the inflammatory mucosa into proximal sites (transverse, and ascending colons, and cecum). We also used the Mayo endoscopic subscore to evaluate the endoscopic appearance where the colonoscopic biopsy was taken [21]. Each score was based on the endoscopic findings, as follows: 0, normal or inactive disease; 1, mild disease (erythema, decreased vascular pattern, and mild friability); 2, moderate disease (marked erythema, absent vascular pattern, friability, and erosions); and 3, severe disease (spontaneous bleeding and ulcerations). This cohort was recruited from our study investigating the association between promoter DNA methylation and clinical phenotypes, [22] host genetic factors [23] and telomere length [24].

### CpG methylation analysis by bisulfite pyrosequencing

DNA was extracted from the specimens by protein precipitation methods. Bisulfite-treated genomic DNA was used to evaluate the methylation status of 5 CpG island promoters by bisulfite pyrosequencing (*NEUROG1*, *CDX1*, *miR-1247*, *CDH1*, and *CDH13*). These genes have been reported to be candidate molecules in EMT. *CDH1*, encoding the transmembrane protein E-cadherin, has been known as an EMT biomarker [9], [10]. A recent genome-wide association scan also identified *CDH1* as the susceptibility locus for UC [17]. *CDH13* also encodes another cadherin protein, H-cadherin, which has been shown to be methylated in colorectal cancer [18]. We also included three CpG island promoters (*NEUROG1*, *CDX1* and *miR-1247*). These genes were recently identified with candidate methylated CpG island promoters using an EMT model with the ectopic expression of the transcription factor Twist1 in immortalized human mammary epithelial cells [20]. We also evaluated the methylation status of the *LINE1* repetitive element, an indicator of global hypomethylation [25]. The bisulfite treatment of DNA was performed with an EpiTect bisulfite kit (Qiagen) according to the manufacturer's protocol. Pyrosequencing was carried out using a PSQ96 system with a Pyro-Gold reagent kit (QIAGEN), and the results were analyzed using PyroMark Q96 ID software version 1.0 (QIAGEN). The primers used for pyrosequencing are listed in Table 1.

**Table 1:** Primer sequences used in pyrosequencing.

Assay name	Forward primer (1st and 2nd step PCR) sequence	Reverse primer (1st step PCR) sequence	Reverse primer (2nd step PCR) sequence	Sequencing primer sequence
*LINE1*	TTTTGAGTTAGGTGTGGGATATA	AAAATCAAAAAATTCCCTTTC	U-AAAATCAAAAAATTCCCTTTC	GGGTGGGAGTGAT
*NEUROG*	TTTGGAGAAGTTTTGGTTAGTTTAGTT	ACCCCCCAATATTTACATAATTTATACTC	U-ACCCCCCAATATTTACATAATTTATACTC	GAGAAGTTTTGGTTAGTTTA
*CDX1*	GTAGAGGAGGTTTTAGGGTTTAGTAT	CCAAACCCAAACTAACTAACCTA	U-CCAAACCCAAACTAACTAACCTA	GTTATGTGTTGGATAAGGAT
*miR1247*	AGATAGTGTTTAGGGTAGTTTAGTTTTTTAGA	AAATCTCCTTTCCCCTTAAACTACAAT	U-AAATCTCCTTTCCCCTTAAACTACAAT	TTTTTTAGAAGGGAGATAGA
*CDH1*	TTTGATTTTAGGTTTTAGTGAGT	ACCACAACCAATCAACAA	U-ACCACAACCAATCAACAA	TAGTAATTTTAGGTTAGAGG
*CDH13*	GYGAGGTGTTTATTTYGTATTTGT	AACCAACTTCCCAAATAAATCAAC	U-AACCAACTTCCCAAATAAATCAAC	TGTTATGTAAAAYGAGGG

U =  biotin labeled universal primer tag: 5′-biotin-GGGACACCGCTGATCGTTTA.

### Statistical analysis

For the paired inflammatory and non-inflammatory colonic mucosa derived from ten patients, an unsupervised hierarchical clustering analysis was used to identify distinct subgroups based on the methylation status of 5 CpG island promoters. The methylation levels between two and three groups were compared using the t-test and Kruskal-Wallis test, respectively. The correlation between the methylation levels of CpG island promoters and the *LINE1* repetitive element, age and duration was assessed using a Spearman correlation analysis. The methylation levels of the CpG island promoters and the Mayo endoscopic subscores were assessed using one-way ANOVA. A *p* value <0.05 was considered statistically significant.

## Results

### Methylation status of EMT related genes among paired samples

**Figure 1:**
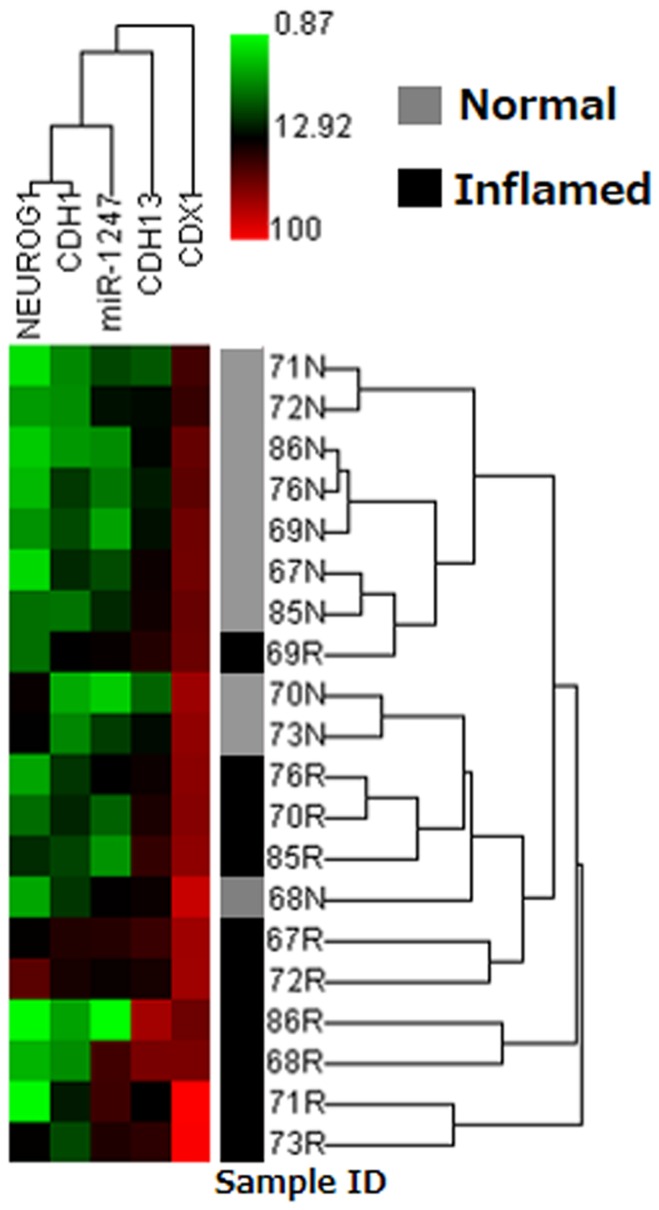
Unsupervised hierarchical clustering analysis using paired inflammatory and non-inflammatory colonic mucosa derived from ten patients. Black boxes, inflammatory rectal mucosa (R); grey boxes, normal proximal mucosa (N); Samples of the same ID number were obtained from the same patients.

Fig. 1 shows the results of an unsupervised hierarchical clustering analysis using paired inflammatory and non-inflammatory colonic mucosa derived from ten patients. This analysis revealed that a majority of the inflammatory rectal mucosa was clustered as hyper methylated samples compared with the non-inflammatory proximal mucosa. One inflammatory sample (69R) was clustered as a relatively hypo methylated sample, and one non-inflammatory proximal mucosa (68N) was also clustered as a relatively hyper methylated sample. However, compared with other samples from the same patients, the inflammatory rectal samples showed hyper methylation in both cases. Therefore, hyper methylation was observed in inflammatory rectal samples compared with the non-inflammatory proximal mucosa in all ten cases. Among all genes tested, we also observed that methylation of *CDX1* was considerably higher compared to other genes (*NEUROG1*, *miR-1247*, *CDH1*, and *CDH13*) across all the samples, possibly due to the difference of methylation susceptibility in colonic tissue or UC. Association between methylation status of EMT related genes and global hypomethylation.

**Figure 2:**
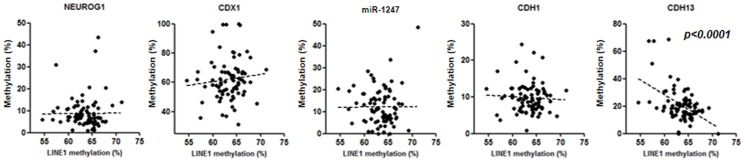
Association between methylation status of EMT-related genes and global hypomethylation assessed by methylation of the *LINE1* repetitive element. Statistical analysis was performed using the Spearman correlation analysis.

To determine the association between the methylation status of EMT related genes and global hypomethylation, methylation of the *LINE1* repetitive element was evaluated. Among all 5 genes, methylation of the *CDH13* promoter was significantly correlated with hypomethylation of the *LINE1* repetitive element (*p*<0.0001), while no association was found with all the other CpG island promoters (all p values>0.1) (Fig. 2).

### Association between methylation status of EMT related genes and clinical phenotypes of UC

**Figure 3:**
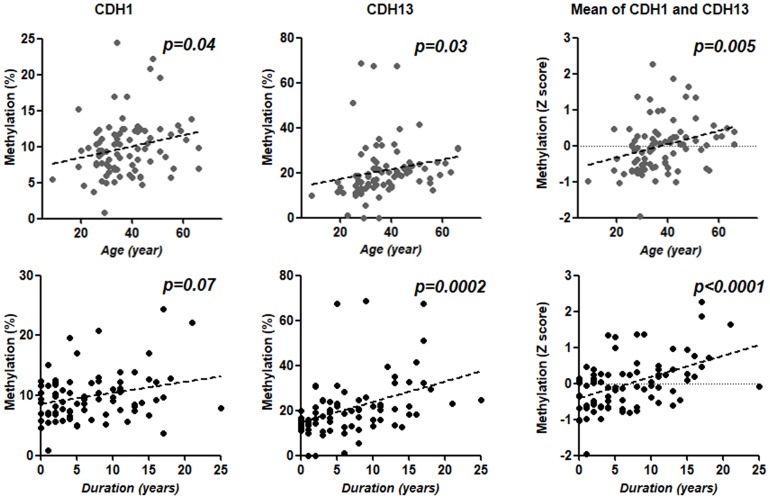
Methylation of *CDH1* (left), *CDH13* (center) and mean Z score of the two genes (right) in relation to the age and duration of disease. Statistical analysis was performed using a Spearman correlation analysis.

To evaluate the association between the methylation status of EMT related genes and clinical UC phenotypes, age, duration of disease, location of inflammation, clinical course, number of hospitalizations, steroid dependency, refractory phenotype and history of surgery were included in the analysis. Of 5 CpG sites, methylation of the *CDH1* and *CDH13* promoters was significantly associated with age (*p* = 0.04, 0.03, respectively). Methylation of *CDH1* was weakly associated with the duration of disease (*p* = 0.07). Methylation of *CDH13* was more closely associated with the duration of disease (*p* = 0.0002). The mean Z score of those 2 CpG promoters was also significantly associated with the duration of disease, and this association was stronger than those with age (*p*<0.00001 *vs.* 0.005) (Fig. 3).

**Figure 4:**
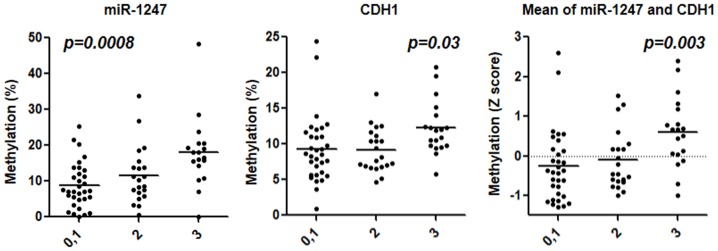
Methylation of *miR-1247* (left), *CDH1* (center) and mean Z score of the two genes (right) in relation to the Mayo endoscopic subscore. The statistical analysis was performed using one way ANOVA.

No significant association was found between the methylation status of 5 genes and the location of inflammation, clinical course and number of hospitalizations. On the other hand, several positive associations were found between the hypermethylation of several genes and more severe UC clinical phenotypes (Table 2 and Fig. 4). For example, the hyper methylation of *CDX1* and *miR-1247* were significantly associated with a refractory UC phenotype (*p* = 0.04 and 0.006, respectively.). A similar trend was found between *CDH1* hyper methylation and the same phenotype (*p* = 0.08). *miR-1247* and *CDH1* hyper methylation were also weakly correlated with steroid dependency (*CDH1*: *p* = 0.09) and history of surgery (*miR-1247*: *p* = 0.06). In addition, there was a significant association between *miR-1247* and *CDH1* hyper methylation and a more severe Mayo endoscopic subscore (*miR-1247*: *p* = 0.0008, *CDH1*: *p* = 0.03, mean of both: *p* = 0.003) (Fig. 4).

**Table 2:** Associations between methylation of EMT related genes and subtypes of UC.

Variables (n)	*NEUROG1* (%)	*CDX1* (%)	*miR-1247* (%)	*CDH1* (%)	*CDH13* (%)
*Extension of the inflammatory lesions*					
Proctitis (20)	9.6±2.0	60.9±2.9	11.0±1.9	9.7±0.8	17.9±1.4
Left sided-colitis (25)	7.5±0.7	61.1±2.7	14.5±2.0	9.5±0.8	24.7±3.8
Pancolitis (41)	9.0±1.1	63.5±2.5	11.3±1.2	10.0±0.7	20.0±1.4
*Clinical courses* ¶					
Only one attack (9)	9.2±1.7	64.1±4.2	10.7±2.9	8.6±0.9	16.2±1.1
Relapse and remitting (38)	7.8±0.9	62.5±2.1	12.8±1.5	10.1±0.6	20.9±2
Chronic continuous (38)	9.5±1.4	61.6±2.7	11.9±1.3	9.8±0.8	22.2±2.3
*Number of times hospitalized*#					
0∼1 (60)	8.6±0.9	61.4±1.8	11.3±0.9	9.6±0.5	21.6±1.7
2∼ (23)	9.6±1.3	63.1±3.1	13.7±2.3	10.4±1.0	19.7±2.3
Steroid dependency $					
No (67)	8.3±0.8	61.3±1.7	11.9±1.1	9.4±0.4	20.2±1.4
Yes (18)	10.5±1.6	66.4±3.7	13.2±1.9	11.3±1.3	24.3±4.0
Refractory &					
No (55)	8.1±1.0	59.9±1.7	10.3±0.9	9.3±0.5	20.9±1.8
Yes (30)	10±1.1	66.8±3.0	15.6±1.9	10.9±0.8	21.1±1.9
Needing surgery*					
No (80)	8.4±0.8	61.9±1.6	11.6±0.9	9.7±0.5	21.5±1.4
Yes (6)	13.0±2.3	66.1±5.4	18.5±6.8	11.5±1.7	13.0±4.1

Note: Data are expressed as the mean ± SE. n, number of samples.

Two and three groups were compared using the t-test and Kruskal-Wallis test, respectively.

¶ ? #, $, & and *: Data are missing for one, three, one, and one cases, respectively.

$: *CDH1*, *p* = 0.09, &: *CDX1*, *p* = 0.04, *miR-1247*, *p* = 0.006, *CDH1*, *p* = 0.08.*: *miR-1247*, *p* = 0.06.

**Figure 5:**
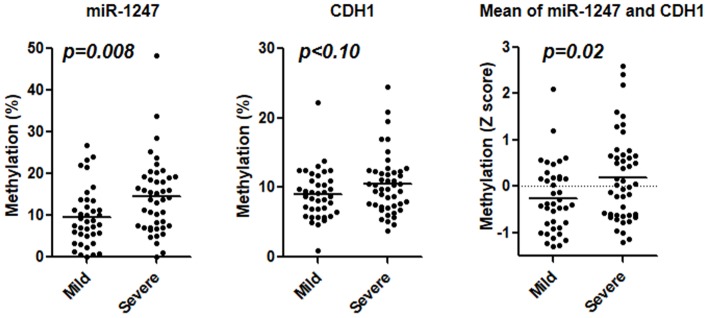
Methylation of *miR-1247* (left), *CDH1* (center) and mean Z score of the two genes (right) in relation to severe clinical phenotype of UC. The statistical analysis was performed using Student's t-Test.

To further investigate the association between the methylation status of EMT related genes and clinical UC phenotypes, a severe clinical phenotype was defined as having any of following phenotypes: hospitalized more than twice, the highest Mayo endoscopic subscore, steroid dependence, refractory disease, or history of surgery. These results demonstrated that *miR-1247* hypermethylation was significantly associated with severe clinical phenotype (*p* = 0.008), and a similar trend was observed for *CDH1* hyper methylation (*p*<0.1). The mean Z score of *miR-1247* and *CDH1* hyper methylation was also associated with the same phenotype (*p*<0.02) (Fig. 5).

## Discussion

Our data show that rectal inflammatory mucosa in UC is characterized by hyper methylation at promoter CpG islands in EMT-related genes. Previous studies have revealed that accelerated DNA methylation is observed in both inflamed and dysplastic colonic epithelium of UC patients, especially in the age-related genes. Our result added the evidence that this methylation is also occurring in EMT-related genes. Recent studies suggest that EMT contributes to the pathogenesis of IBD [13], [14]. One good example is reduced expression of *CDH1* encoding the epithelial cell marker E-cadherin, which may be partly explained by promoter CpG island methylation [15]. *CDH13*, encoding another cadherin protein H-cadherin, has also been reported to be methylated in colorectal cancer [18], an established complication of longstanding ulcerative colitis [19]. In this study, we also examined new candidate genes (*NEUROG1*, *CDX1* and *miR-1247*) that have recently been identified with candidate methylated CpG island promoters using an EMT human cell line model [20]. Several studies also indicate that methylation of *NEUROG1*, *CDX1* and *miR-1247* are involved in carcinogenesis in the colorectal (*NEUROG1*, *CDX1*) [26], [27] and stomach (*miR-1247*) [28]. In paired colonic samples from UC patients, the inflamed rectal mucosa were well separated from the normal-appearing mucosa using the methylation status of 5 candidate genes by an unsupervised hierarchical clustering analysis. It can be speculated that the accumulation of promoter CpG island methylation of EMT-related genes affects gene expression, leading to a disturbance of intercellular adhesion in the intestinal epithelium in UC.

In inflamed and neoplastic epithelium, promoter CpG hyper methylation is closely correlated with global hypomethylation [24]. However, such an association was observed only in the *CDH13* promoter, but not for the remaining four genes, suggesting that the methylation of a majority of EMT-related genes is not linked to global hypomethylation.

We have also shown that the hypermethylation of EMT-related genes is associated with clinical UC phenotypes. The *CDH1* and *CDH13* promoters were significantly associated with patient age. Similarly, the methylation of those genes is correlated with the duration of disease, which is a well-accepted risk factor for cancer occurrence. Several positive associations were also found between the hypermethylation of EMT-related genes and more severe clinical UC phenotypes (hypermethylation of *CDX1* and *miR-1247* and refractory phenotype, hypermethylation of *miR-1247* and *CDH1* and severe Mayo endoscopic subscore). The mean Z score of *miR-1247* and *CDH1* hyper methylation was significantly associated with a severe clinical phenotype (more frequent hospitalization, the highest Mayo endoscopic subscore, steroid dependence, refractory disease, or history of surgery).

Our data suggest that there may be more variability in methylation status in the EMT-related genes during inflammation that can discriminate several phenotypic differences. UC is extremely heterogeneous in its clinical course, prognosis, and response to treatment; therefore, it is hypothesized that UC is a syndrome in which different pathogenic mechanisms lead to various clinical phenotypes, and it may be necessary to place greater emphasis on disease heterogeneity. Because severe phenotypes require more intensive clinical treatment, the results suggest that different UC subgroups have different molecular backgrounds. Our observation of an association between the hyper methylation of EMT-related genes and severe clinical UC phenotypes in the easily accessible rectum also indicates these genes' potential usefulness as molecular markers to direct more appropriate therapy that reflects an individual's pathophysiology. The findings also suggest that chronic inflammation in UC eventually leads to cancer progression [1]. Because severe inflammation characterized as having more frequent hospitalization, the highest Mayo endoscopic subscore, steroid dependence, refractory disease, or history of surgery would finally confer greater risk for developing cancer in UC, it is possible that hypermethylation predicts populations at high risk for developing colorectal cancer, which was not directly evaluated in this study. Since methylation of all genes examined have been reported to have roles in carcinogenesis in digestive tract, including colorectal and stomach. A well-designed prospective study will be needed to further clarify the clinical importance of DNA methylation in EMT-related genes as a biomarker for risk assessment of colorectal cancer in UC patients.

One limitation which should be noted is that the detailed mechanisms of how these EMT-related genes contribute to the pathogenesis of UC and/or UC-related carcinogenesis is unclear. More intensive experiment will be needed to examine the methylation of EMT-related genes in UC from the mechanistic view point including the interaction with gene expression and histone modifications etc.

Moreover, it is shown that differences in cell or tissue population will influence the nature of DNA. Since the majority of change with disease progression could occur in the epithelial DNA, it would be ideal to separate or enrich epithelial DNA from tissue. From the histological assessment from adjacent to mucosa from biopsy for DNA extraction, we confirmed that all the biopsies contained at least more than 70% of epithelial cells, however, it was not exactly same specimens used for DNA extraction. It should be therefore noted that result might be influenced depending on the difference of cell or tissue population and the methods to isolate colonic epithelium need to be considered for the future study.
